# Road Extraction with Weak Features and Complex Backgrounds Based on Atrous–Strip–UNet

**DOI:** 10.3390/s26041134

**Published:** 2026-02-10

**Authors:** Yanni Ma, Junchuan Yu, Yuxiu Hao, Yangyang Chen, Yu Wang, Qiong Wu, Yuanbiao Dong, Dawei Sun

**Affiliations:** 1 School of Artificial Intelligence, China University of Geosciences (Beijing), Beijing 100083, China; mayanni@mail.cgs.gov.cn; 2 China Natural Resources Aerial Geophysical and Remote Sensing Center, Beijing 100083, China; 3 School of Computer and Communication Engineering, Beijing University of Science and Technology, Beijing 100083, China

**Keywords:** road, remote sensing, strip convolution modules, ASUnet

## Abstract

**Highlights:**

**What are the main findings?**

- An atrous–strip–unet (ASUNet) is proposed, which integrates atrous convolution and strip convolution modules into an encoder–decoder architecture to address road extraction challenges from remote sensing images (e.g., weak road features, complex backgrounds, and occlusions).
- On the self-compiled Zhouqu Road Dataset (covering rural/suburban/urban roads of western Chinese counties) and the public DeepGlobe Dataset, ASUNet achieves F1 scores of 0.7292 and 0.7134, respectively. The proposed algorithm demonstrates high accuracy and effectiveness for road extraction, constituting a valuable addition to the road extraction toolkit.

**What are the implications of the main findings?**

- ASUNet provides a reliable approach for accurate road extraction in remote sensing scenarios with weak features or complex backgrounds, supporting applications like transportation planning and disaster emergency response.
- The Zhouqu Road Dataset complements the lack of representative county-level road data in western China, offering a valuable resource for subsequent remote sensing road extraction research.

**Abstract:**

With the continuous improvement of remote sensing image resolution, accurately extracting road information from complex backgrounds remains challenging. This is because roads present diverse morphological characteristics across regions and scales, and their spectral features are highly similar to those of surrounding objects, such as buildings and bare soil, making them hard to distinguish. Occlusion by buildings and trees leads to incomplete road extraction. To solve the above problems, this paper proposed the atrous–strip–Unet (ASUNet), an encoder–decoder network into which atrous and strip convolution modules are inserted to extract roads with weak features and complex backgrounds from high-resolution remote sensing images. In this study, we construct the Zhouqu Road Dataset from high-resolution aerial imagery, covering representative road types (rural, suburban, and urban) characteristic of county-level settlements in western China. By comparing several advanced algorithms with excellent learning performance—including BiSeNet and LinkNet—on both the Zhouqu Road and DeepGlobe Datasets, the improved and optimized model presented in this paper demonstrates better extraction accuracy and effectiveness; it achieves F1 scores of 0.7292 and 0.7134 on the two datasets, respectively. It is particularly worth mentioning that our proposed algorithm shows better performance in scenarios where road features are weak or backgrounds are complex.

## 1. Introduction

Road information extraction from remote sensing images has enormous potential in various fields such as disaster response, autonomous driving, urban planning, and intelligent transportation. The continuous improvement of satellite optical image resolution and the rapid development of computer vision have made accurate road extraction possible. However, factors such as vegetation, shadows, diverse land-cover types, and blurry imaging edges result in significant differences in the appearance of different road types, making accurate extraction work more complex. Given the sheer volume of data, accurate road extraction remains a challenging and vital research topic [1].

Essentially, road extraction from remote sensing imagery constitutes a specialized instance of semantic segmentation. Accordingly, this paper structures its review along the evolutionary trajectory of remote sensing image-analysis techniques. Prior to the deep learning era, road extraction (or remote sensing image information extraction) went through three stages: The first stage is the integration of prior knowledge with expert interpretation and expert recognition [2]. This method can accurately extract road boundaries, but it requires a significant amount of manpower and resources. The second stage is the use of pixel-based classification methods that utilize the grayscale values of images. This method is difficult to apply in complex environments with various interference factors, such as vehicles, trees, and buildings, and requires a lot of post-processing [3]. The third stage is the use of object-oriented methods, which integrate the spectrum, texture, structure, and topological relationships of roads as a whole to improve efficiency and accuracy [4]. This method has significantly transformed the approach to road extraction; however, it is prone to classification errors when dealing with spatially adjacent objects that share similar shapes. When extracting different types of roads, the issue of scale is particularly important [5], and it is necessary to adjust and test parameters to obtain relatively good results.

In recent years, the rapid development of deep learning technology has completely changed computer vision and ushered in a new era of remote sensing information extraction. Deep learning-based methods autonomously learn complex road features from large datasets without human intervention. Such methods, stemming from a common core approach, perform well in gradually extracting semantic information from raw data. Compared with traditional algorithms, deep learning methods offer higher efficiency and effectiveness, thus becoming the preferred methods for road extraction tasks [6,7]. In terms of the semantic segmentation framework, Long et al. [8] proposed the Fully Convolutional Network (FCN), which laid the foundation for pixel-level classification. UNet, proposed by Ronneberger et al. [9], achieves fine boundary localization under small sample conditions through a symmetric encoding–decoding structure. Subsequently, DeepLab [10] introduced dilated convolution and fully connected CRF to enhance edge consistency. To address the issue of insufficient topological connectivity on roads, Mosinska et al. [11] jointly optimized pixel loss and topological perception loss, while Batra et al. [12] further improved the ability of the network to retain slender structures through directional field learning. Driven by the demands of computational efficiency and real-time performance, Chaurasia et al. [13] proposed a lightweight LinkNet, while Cheng et al. [14] designed a cascaded high-efficiency Cascaded Efficient (CE) RoadNet to achieve rapid inference at the city level with extremely low parameter count. The untrained Res-U2Net [15] further pushes the lightweight idea to its limit by completely removing the need for training data.

For the diversity of road types and spatial scales, research presents a parallel evolution pattern of “refinement lightweight large-scale”. Regarding the improvement of geometric accuracy in urban scene focusing, Zhang et al.’s Residual U-Net [16], Zhong et al.’s direction enhance- Feature Enhancement of Road Directionality Network (FERDNet) [17], and Zhu et al.’s Swin Transformer structure continuously refresh boundary fidelity on high-resolution images [18]. The Receptive Field-YOLOv5 (RE-YOLOv5) proposed by Li et al. [19] significantly expands the receptive field and suppresses background interference through a plug-and-play multi-branch hollow receptive field (RFE) module and channel space dual attention, achieving high recall detection of severely occluded road targets with zero additional computational cost. The Transferable Contextual Network (TCN) proposed by Wang et al. [20] utilizes global local context aggregation and domain alignment to transfer pretrained urban weights to UAV images without relying on any rural annotations, achieving accurate extraction of rural roads. In forests and remote areas, Trier and Salberg [21] and Sloan et al. [22] respectively use CubeSat, Sentinel-2, and general satellite imagery to achieve automatic discovery of newly built or existing forest trails at the regional to national scale, supporting forest management and ecological protection. The above research further confirms the wide applicability of deep learning methods in urban–suburban road extraction [4,7].

To achieve vectorized representation of large-scale road networks, Bastani et al. [23] proposed RoadTracer, which directly generates vector road networks through the iterative search of convolutional neural network (CNN) decision functions, significantly improving the completeness and accuracy of urban-level road network reconstruction. At the same time, multi-source data fusion and post-processing techniques continue to expand the deep application of road information. Huang et al. [24] used an improved CycleGAN to restore Ground Penetrating Radar (GPR) images, providing supplementary information for the detection of complex road surface structures in cities. The Multi-hierarchical Cross Transformer Network (MCT-Net) fusion of hyperspectral and multispectral images by Wang et al. [25] can provide richer spectral spatial feature sources for road extraction. Zhou et al. [26] proposed the direction-guided post-processing network Rirnet to infer more complete and coherent road information. Yuan et al. [27] expanded the application scenarios of road extraction results in urban computing by segmenting urban functional areas based on road networks.

In summary, current research has formed multiple technical routes, such as “CNN Transformer hybrid architecture, topology constraints, directional priors, lightweight design, and multi-source fusion” [28,29,30], but further balancing accuracy, connectivity, and computational efficiency remains necessary in large-scale complex scenarios.

Despite a series of advancements in road detection, numerous challenges remain. To summarize: (1) The proportion of roads is relatively low, resulting in a serious imbalance between the foreground and background in the image. (2) When extracting rural roads, the shape of a road is very similar to land types, such as rivers and gullies, and the information of roads with small width or length in remote sensing images is weak [30], so it is easy to lose the information of small roads during the feature extraction process, resulting in the omission of small roads [27]. (3) In urban roads, the scene is more complex and obstructed by vehicles, buildings, and trees, making it very difficult to extract continuous roads.

In this paper, the Atrous–Strip–UNet (ASUNet) model is employed to learn road features. The model adopts an encoder–decoder architecture, which is further optimized by combining the atrous convolution module with a wide field of view and the strip convolution module, which is more similar to road features, to extract road information from remote sensing satellite images.

## 2. Method

In the field of remote sensing image analysis for road extraction, this paper proposes an ASUNet network model for generating road semantic segmentation. This method is based on a combination of a deep learning-based neural network architecture and specialized convolution operations. It combines Unet, ResNet50, strip convolution, and atrous convolution, among which strip convolution and atrous convolution are particularly important. Therefore, this article first introduces the overall network structure of Asunet and then provides a detailed introduction to strip convolution and atrous convolution.

### 2.1. ASUnet for Road Extraction

**Figure 1:**
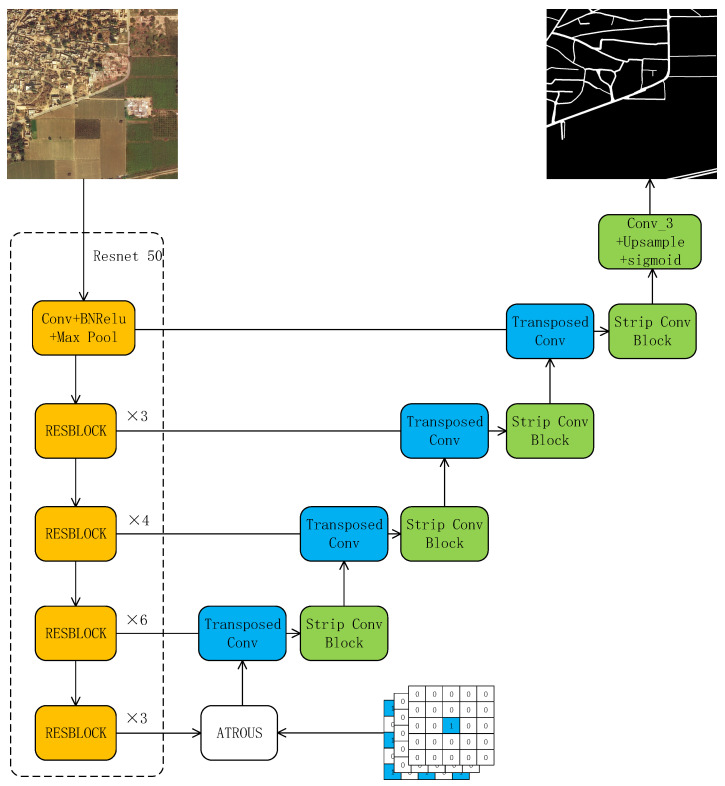
ASUNet network structure.

As shown in Figure 1, for the encoding part, due to its outstanding capability in feature learning, ResNet was chosen as the encoder in this paper, pretrained on ImageNet [31]. The reason why ResNet-50 is selected as the backbone network lies in its core advantage of balancing the performance requirements and engineering practicality of road extraction tasks. With moderate depth, ResNet-50 not only avoids the insufficient feature expression of shallow networks and effectively captures the multi-scale features of roads—from edge details to global semantics—but it is also more lightweight than deeper networks, such as ResNet-101. This makes it suitable for processing scenarios with large amounts of remote sensing image data, balancing extraction accuracy and inference speed. Meanwhile, the residual connections can transmit gradients, addressing the gradient vanishing issue in deep network training and facilitating the capture of subtle differences between roads and backgrounds in complex scenes. Furthermore, its pretrained weights on the ImageNet dataset can be transferred to the road extraction task to accelerate model convergence, and the 2048-dimensional feature output can also carry rich semantic information, reducing misclassification between roads and similar ground objects. The ResNet-50 encoder comprises a series of convolutional layers that perform five stages of downsampling to reduce resolution and increment the receptive field of feature maps.

In the decoding part, the learned features are upsampled to the size of the input image using transposed convolution, and a strip convolution module is utilized to extract linear features of roads. However, relying solely on the encoder’s final output feature map in the decoder risks losing pixel details and missing small roads [32]. To address this, this paper follows the UNet model’s approach by incorporating skip connections, which facilitate the sharing of low-level information between the encoder and decoder. This ensures the retention of low-level features and the fusion of multi-scale features [33]. The feature maps from the first four layers of the encoder, along with the output from the atrous convolution module, are utilized by the decoder. This allows the decoder to extract multi-scale contextual information from high-level feature maps and to recover edge details from low-level feature maps, thereby refining the decoder’s output.

In summary, the proposed ASUnet model, which leverages an encoder–decoder structure network to learn road features, incorporates atrous convolution and strip convolution modules to enhance the model’s performance in extracting roads from remote sensing satellite images. Figure 1 shows the ASUNet network architecture diagram.

### 2.2. Strip Convolution Modules

The majority of convolutional neural networks currently use square convolution kernels to learn feature maps within square windows. This convolution operation is very suitable for most natural objects with block shapes. But the road studied in this paper has the characteristics of a narrow, large span, and continuous distribution [34]. Therefore, using square convolution to capture road features may require a very large square to cover a road, an approach that inevitably merges a lot of irrelevant information. The strip convolution used in this paper is more in line with the shape of the road, as it uses a long strip convolution kernel to capture the features of the road area. Band convolution captures local contextual information along a specified spatial direction and prevents irrelevant regions from interfering with feature learning [35], which is defined as

(1) $$Y_{D}\left[i,j\right]={\left(X\times K\right)}_{D}=\sum_{t=-r}^{r}x\left[i+D_{h}t,j+D_{w}t\right]\cdot K\left[r-t\right]$$

where $Y_{D}\left[i,j\right]$ represents the value at position $\left[i,j\right]$ in the output feature map $Y_{D}$, X means the input image or feature map, r means the radius of the convolution kernel, t means an index variable within the convolution kernel, and $D_{h}$ and $D_{w}$ represent the direction components of the convolution kernel in the horizontal (width) and vertical (height) directions. The elements of set D can take the values (0,1), (1,0), (1,1), and (−1,1). For the convolution kernel K, this paper sets r to 4 so that the strip convolution kernel has 9 parameters, which is the same number of parameters as the 3 × 3 square convolution operation. All strip convolution kernels K are initialized using the normal initialization method proposed by He et al. [36]. For this method, the standard deviation σ is calculated as follows:

(2) $$\sigma =\sqrt{2/({fan}_{out})}$$

where σ denotes the standard deviation of the output signal and ${fan}_{out}$ is the fan-out parameter (the number of output neurons connected to a single input neuron).

**Figure 2:**
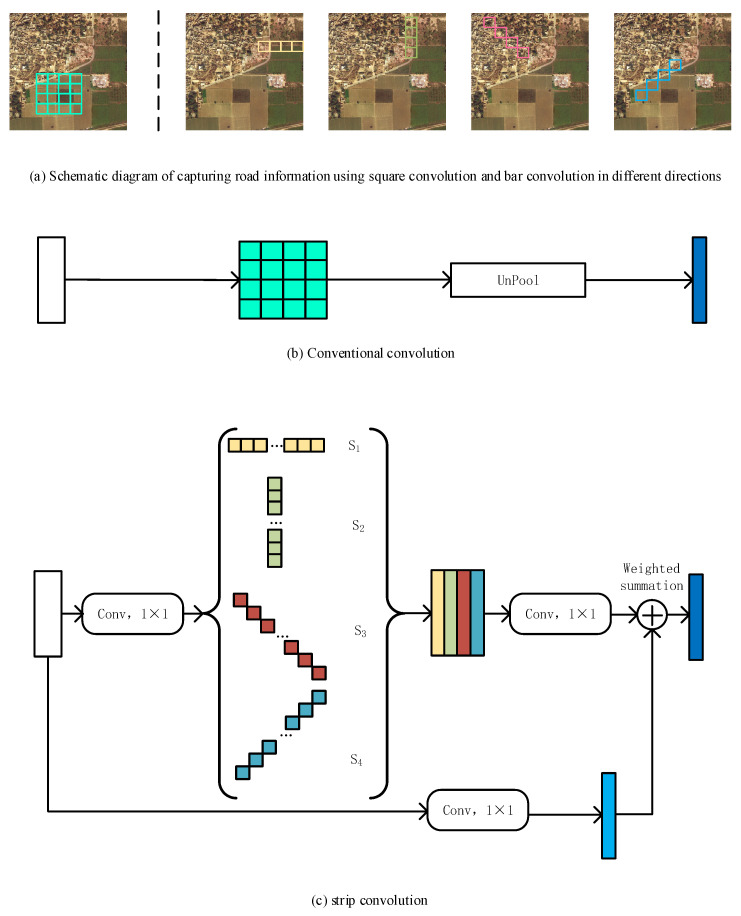
Schematic diagram of the strip convolution module.

As shown in Figure 2, the strip convolution module uses four strip convolutions, namely, horizontal, vertical, left diagonal, and right diagonal, to capture long-range contextual information from four different directions. This allows each pixel in the output feature map to establish connections with multiple pixels in the input feature map in all four directions. This aligns more closely with the linear characteristics of roads.

In the strip convolution module, the input tensor is passed through a 1 × 1 convolution operation and sent to four parallel branches, each branch representing a direction of strip convolution. The four output tensors are then concatenated, followed by a 1 × 1 convolution operation. In order to improve feature reuse, this paper uses residual connections to add the original input tensor to the output tensor while preserving feature information [37].

### 2.3. Atrous Convolution

Atrous convolution, also known as dilated convolution, is a type of convolution operation in convolutional neural networks, initially introduced to address image segmentation problems. Common image segmentation algorithms often use pooling layers to increase the receptive field, but this can also reduce the size of feature maps. In such cases, upsampling is typically used to restore the image size, but this process can lead to loss of details in the features. In contrast, atrous convolution allows for enlarging the model’s receptive field without reducing image resolution. Atrous convolution introduces one or more gaps in the convolutional kernel to increase the receptive field, enabling the network to capture a broader context of information. Compared to regular convolution operations, atrous convolution maintains the same number of parameters, thus avoiding an increase in the model’s parameter count.

**Figure 3:**
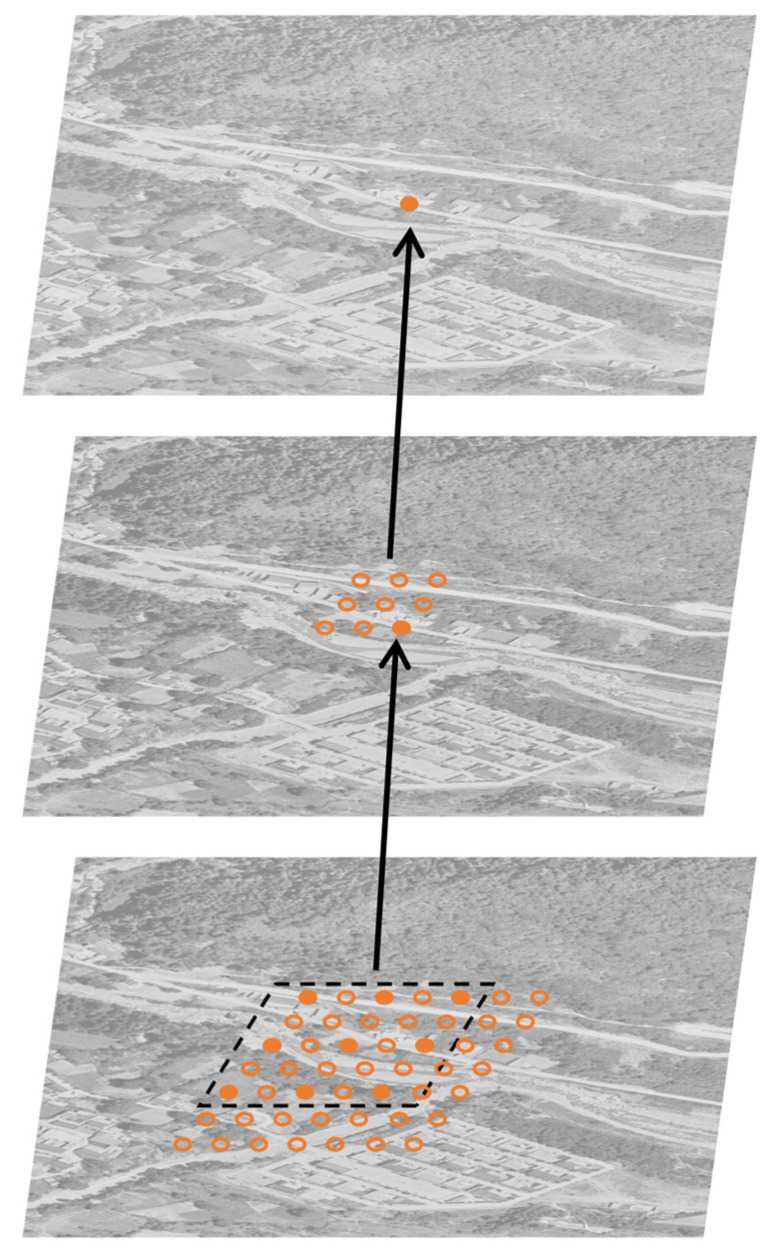
Schematic diagram of atrous convolution.

The receptive field of pixels in the output feature map can be adjusted by modifying the dilation rate (Figure 3). However, it is important to note that the receptive field in deep neural networks is a cumulative result of multiple layers rather than being determined by a single layer. The general recursive formula for calculating the cumulative receptive field across multiple layers is as follows:

(3) $$F_{l}=RF_{l-1}+\left(k_{l}-1\right)\times r_{l}\times \prod_{i-1}^{l-1}s_{i}$$

where $RF_{l}$ is the cumulative receptive field of pixels in the output feature map of the l-th layer, which specifically refers to the size of the region in the original input image that has an impact on a single pixel in the l-th layer’s output feature map; $k_{l}$ is the size of the convolution kernel adopted in the l-th layer. For the first layer (where l = 1), $RF_{l}$ equals $k_{l}$ because there are no preceding layers to contribute to the receptive field; $r_{l}$ is the dilation rate of the convolution operation in the l-th layer, which controls the spacing between the elements of the convolution kernel, allowing the receptive field to expand without increasing the actual size of the kernel; and $s_{i}$ is the stride of the convolution (or pooling) operation in the i-th layer. $s_{i}$ determines the distance by which the convolution kernel (or pooling window) shifts across the input feature map during the computation process [38].

Three parallel atrous convolution layers are employed in this work, featuring dilation rates of 1, 2, and 4 that adhere to the exponential increment pattern of 2^i^ (i = 0, 1, 2). A 3 × 3 kernel is adopted for all convolutional operations, and the padding value is set equivalent to the respective dilation rate to preserve the feature map size. This architectural design achieves efficient expansion of the receptive field and integration of multi-scale contextual information, without imposing additional computational overhead.

**Figure 4:**
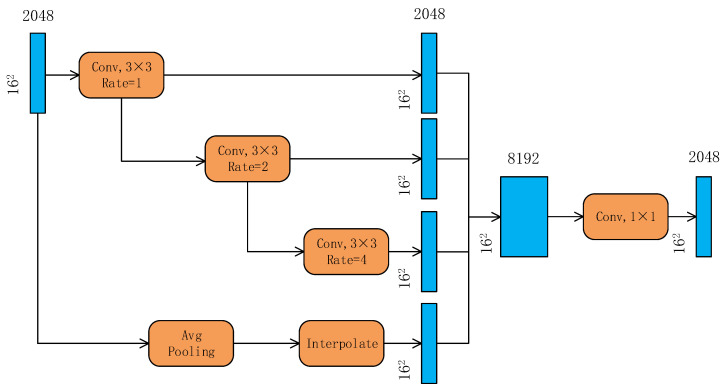
Schematic diagram of atrous convolution module.

The encoder output is rich in advanced features. To better utilize the encoder output, this paper introduces an atrous convolution module at the bottom of the encoder to expand the receptive field. As shown in Figure 4, the atrous convolution module has four parallel branches with different receptive field sizes. The first three branches are fused by cascading to integrate feature maps of different receptive fields, while the last branch introduces image-level features through global average pooling. The number of channels after concatenation is then reduced to the number of input channels using 1 × 1 convolution.

The size of the receptive field in the feature map can be adjusted by changing the dilation rate. However, there is a grid effect in the atrous convolution, which leads to the loss of local information and decreased performance in detecting small targets. Cascading atrous convolutions with different dilation rates helps to obtain information from a wider range of pixels and avoid the grid effect. In this paper, ResNet-50 reduces the image size from 512 × 512 to 16 × 16 through five downsamplings. Therefore, the dilation rates of the cascaded convolutions in the atrous convolution module are set to 1, 2, and 4, and the receptive fields of the parallel branches are 3, 7, and 15, respectively, which can roughly cover the entire feature map. On this basis, a global average pooling branch is added to obtain the global information of the image. The formula for the receptive field size of the cascaded atrous convolutions is as follows:

(4) $$S=S_{1}+S_{2}-1$$

where $S_{1}$ and $S_{2}$ are the receptive fields of the two cascaded atrous convolutions.

## 3. Experiments and Analysis

### 3.1. Datasets

**Figure 5:**
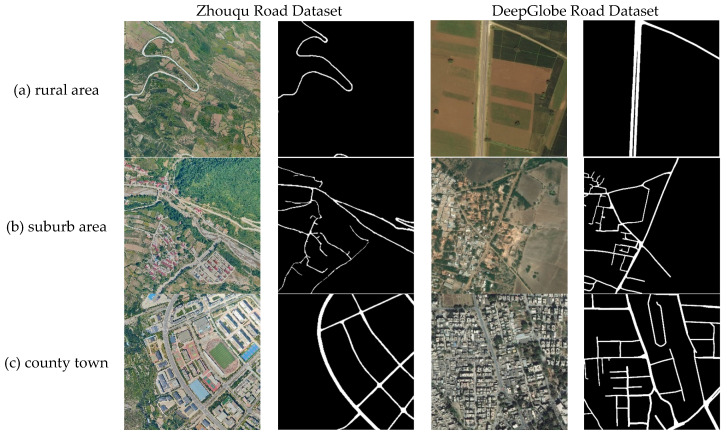
Examples of Zhouqu and DeepGlobe road images.

Two datasets were used in the experiment to verify the accuracy of the model. They are the Zhouqu Road Dataset and the DeepGlobe Dataset, and road image examples from the datasets are shown in Figure 5.

The Zhouqu Road Dataset is an aerial image dataset of Zhouqu, which covers road images in multiple scenes, such as the county town, suburb areas, rural areas, and mountainous areas of Zhouqu County, Gansu Province, China. This dataset can, to some extent, represent the road characteristics of mountainous and hilly counties in central and western China. The roads included in our dataset mostly refer to hardened pavements, including asphalt and cement pavements, as well as a small number of rural roads with soil. Data were captured with a PhaseOne iXU-RS1000 sensor (Phase One, Copenhagen, Denmark) between July and September 2022 at an original ground-sample distance of 0.15 m and later resampled to 1 m. The resulting images measure 1024 × 1024 pixels with a spatial resolution of 1 m; the average road width is 5.55 m. The dataset is annotated with 1305 images. This paper divided the dataset into 910 images for training, 195 images for validation, and 200 images for testing based on an 8:1:1 ratio. The experiment enhanced the training dataset through scaling, rotation, cropping, and other methods.

The DeepGlobe Dataset is a publicly available dataset provided on the official website of the CVPR 2018 Road Extraction Challenge [39]. This dataset covers road images in multiple scenarios, including cities, suburb areas, rural areas, mountainous areas, etc. Moreover, the dataset has a moderate scale and has been widely used and recognized in the academic community. The DeepGlobe Dataset was used in the experiment to evaluate the performance of the proposed method in this paper. The pixel resolution of the images in this dataset is 0.5 m, and the image size is 1024 × 1024 pixels; the average width of the road is 3.58 m. The images were collected from three regions, namely, Thailand, Indonesia, and India. This dataset contains 6226 annotated images. This paper divided the dataset into 4980 images for training, 623 images for validation, and 623 images for testing based on an 8:1:1 ratio. The experiment enhanced the training dataset through scaling, rotation, cropping, and other methods.

For training configuration, the following protocol was adopted on the designated training set: Images were augmented by random rotation, horizontal flipping, and Gaussian blur. All inputs were resized to 512 × 512 pixels to guarantee consistency. The initial learning rate was set to 1 × 10^−3^, and optimization was performed with the Adam optimizer coupled with a weight-decay coefficient of 1 × 10^−5^ to mitigate overfitting. Training was run for 100 epochs, employing Focal Loss throughout to counteract class imbalance.

### 3.2. Evaluation Metric

1. McNemar test.

The McNemar test [40] is a statistical test used on paired nominal data, and it can be used to compare the classification results obtained with two methods. A 2 × 2 contingent matrix, denoted by M, was established for this comparison, and it was calculated as follows:

(5) $$M=\left[\begin{array}{c} f_{11}\;f_{12}\;\\ {\;f}_{21\;}\;f_{22\;} \end{array}\right],\;\chi^{2}=\frac{{\left(f_{12}-f_{21}\right)}^{2}}{f_{12}+f_{21}}\;$$

where $f_{11}$, $f_{12}$, $f_{21}$, and $f_{22}$ are the number of pixels classified as follows: classified correctly in both methods, the first one correctly but the second one incorrectly, the first one incorrectly but the second one correctly, and neither correctly, respectively. The $\chi^{2}$ statistic is derived by squaring the difference between the discordant error counts of the two models and dividing by the total number of discordant pairs, thereby quantifying the extent to which the observed discrepancy deviates from the random fluctuation expected under the null hypothesis of equivalent classifier performance; larger values indicate that the difference is increasingly unlikely to be attributable to chance.

2. Evaluation indicators.

Six commonly used evaluation metrics in semantic segmentation are employed as experimental evaluation metrics, namely, accuracy, precision, recall, F1 score, intersection over union (IoU), and mean intersection over union (mIoU) [24]. The calculation formulae for the six evaluation indicators are as follows:

(6) $$Accuracy=\frac{TP+TN}{TP+TN+FP+FN}$$

(7) $$Precision=\frac{TP}{TP+FP}$$

(8) $$Recall=\frac{TP}{TP+FN}$$

(9) $$F1=\frac{2\times Precision\times Recall}{Precision+Recall}$$

(10) $$IoU=\frac{TP}{TP+FP+FN}$$

(11) $$mIoU=\frac{1}{n}\sum_{i=1}^{n}IoU_{i}$$

where TP, TN, FP, and FN represent correctly classified road pixels (true examples), correctly classified background pixels (true counterexamples), incorrectly classified road pixels (false positive examples), and incorrectly classified background pixels (false counterexamples), respectively [41].

### 3.3. Comparison with Other Methods

In this section, we will compare ASUNET with several other advanced road methods, including BiSeNet [42] and LinkNet networks [26], and conduct experimental comparisons using the same datasets, namely, the DeepGlobe Dataset and the Zhouqu Road Dataset. The reason for choosing these two methods for comparison is that they are both based on semantic segmentation frameworks but have their own focuses on network structure, design goals, and applicable scenarios, which can be seen as evolutionary paths from general segmentation to road-specific optimization.

LinkNet introduces dilated convolution in the bottleneck layer to expand the receptive field and enhance the modeling ability for slender and continuous road structures. As a specialized optimized version of UNet for road extraction tasks, it solves the problem of insufficient receptive field in the central layer of standard UNet. BiSeNet adopts a dual path structure: one path extracts spatial details (shallow features), and the other extracts semantic information (deep features), suitable for initial screening of large-scale expressways. ASUnet is a fine segmentation network that combines hollow convolution modules and strip convolution modules to improve the accuracy of road boundaries in complex scenes and solve the problem of breakage caused by occlusion, shadows, and other factors. It suppresses background noise (such as buildings and trees) through attention mechanisms to enhance focus on road areas. Regarding logical positioning, this model is LinkNet’s refined version, suitable for high-precision road extraction tasks. All three methods adopt the encoder–decoder paradigm of UNet but optimize the continuity, real-time performance, and anti-interference of road extraction through different mechanisms (dilated convolution, lightweight structure, attention), forming a logical evolution chain from general to specific and from rough to fine. Therefore, choosing this method for comparison is more targeted.

To analyze the significance of classification accuracy of different algorithms, McNemar tests [38] were performed on the prediction results of ASUnet, LinkNet, and BiSeNet models on the Zhouqu Dataset and DeepGlobe Dataset, pairwise. The inspection results are shown in Table 1. The X^2^ values of the McNemar test for each combination feature were all greater than 10.83 (the critical value of *p* < 0.001), and all passed the significance test. On the Zhouqu Dataset, compared to BiSeNet, ASUNet provides an additional 2.63 M pixels of accuracy (≈ +1.26%). Compared to LinkNet, ASUNet has an additional 389 k pixels of accuracy (≈ +0.19% absolute pixel accuracy). LinkNet also has 2.24 million more correct pixels than BiSeNet (≈ +1.08%). On the DeepGlobe Dataset, compared to BiSeNet, ASUNet provides an additional 18 M pixels of accuracy (≈ +4.5%). Compared to LinkNet, ASUNet has an additional 14 M pixels of accuracy (≈ +3.4% absolute pixel accuracy). LinkNet also has 13 M more correct pixels than BiSeNet (≈ +3.3%). The road extraction results of the models show significant differences among each other, and ASUNet has the highest pixel-level accuracy in the Zhouqu road extraction task.

Due to the serious issue of class imbalance in road extraction, F1 score, road IoU, and mIoU evaluation metrics can better reflect the performance of the model. For the two road segmentation datasets, the Zhouqu Road Dataset and the DeepGlobe Road Dataset, the ASUnet model has shown significant performance advantages: on the Zhouqu Road Dataset, compared with BiSeNet and LinkNet, ASUnet has improved F1 scores by 0.2065 and 0.0287, respectively, and mIoU scores by 0.1211 and 0.0223, respectively; on the DeepGlobe Road Dataset, compared with BiSeNet and LinkNet, its F1 scores have increased by 0.0652 and 0.0314, respectively, and its mIoU scores have risen by 0.0415 and 0.0209, respectively. Overall, ASUnet performs well in terms of accuracy, precision, recall, F1 score, road IoU, and mIoU evaluation metrics. Table 2 lists the experimental results of the evaluation model.

**Figure 6:**
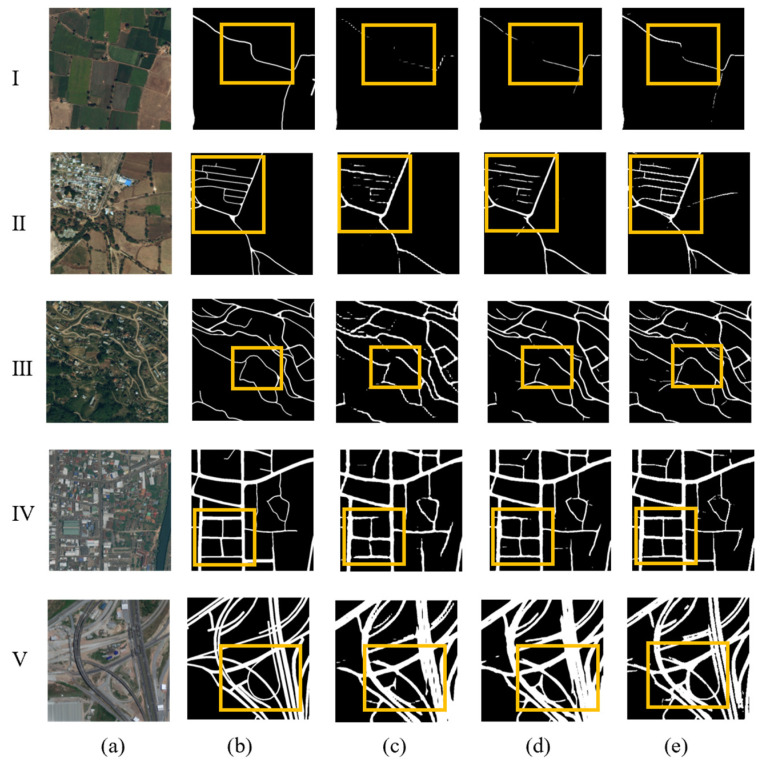
Comparison of experimental results for the DeepGlobe test set. (**a**) Remote sensing imagery; (**b**) road labels; (**c**) BiSeNet; (**d**) LinkNet; (**e**) ASUnet; I, II: rural area; III: suburb area; IV, V: county town.

Figure 6 shows the visualization experimental results of the DeepGlobe Road Dataset. It can be seen that several methods (BiSeNet, LinkNet, ASUnet) can effectively extract roads from complex backgrounds. Compared with these methods, we pay more attention to the road information within the yellow rectangle range, which generally has the characteristics of weak road information that is easily overlooked or affected by vegetation and building obstruction. Specifically, narrow roads are small in size and surrounded by vegetation (I), and the road information is similar to the background information of surrounding buildings (II, IV). Neither of the control models can effectively detect these roads, but the model in this paper can extract them, and the extracted information has stronger continuity. For the case where the difference between farmland information and road information is small (III), this model can effectively avoid missing extraction and extract more coherent information. For complex urban roads (V), the model can also extract road information in complex scenes, but for more special road types, such as overpasses, there is still room for improvement in the information provided due to their extremely small sample size.

**Figure 7:**
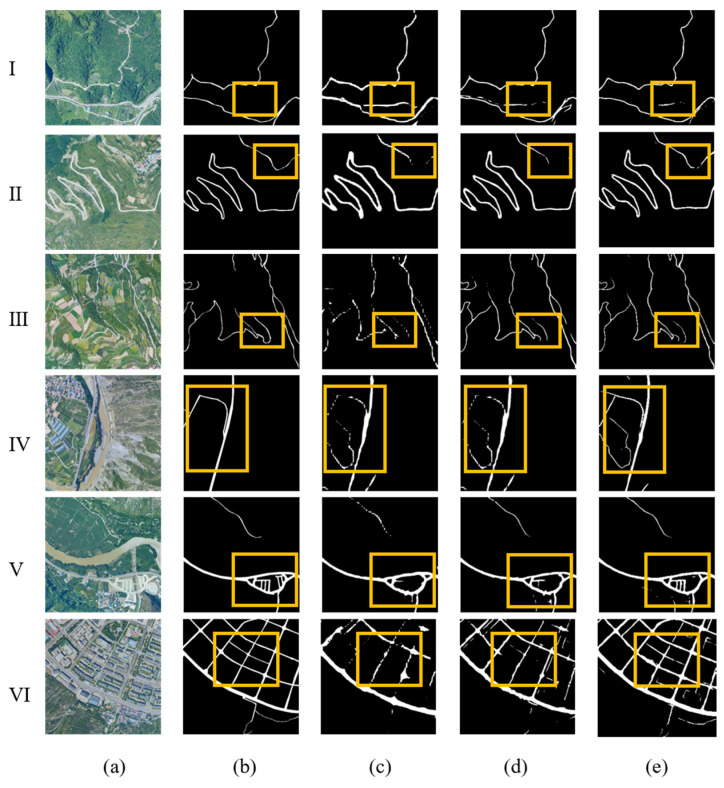
Comparison of experimental results for the DeepGlobe test set. (**a**) Remote sensing imagery; (**b**) road labels; (**c**) BiSeNet; (**d**)LinkNet; (**e**) ASUnet; I, II, III: rural area; IV, V: suburb area; VI: county town.

Figure 7 shows the comparison between different algorithms on the Zhouqu Road Dataset. Several methods have shown good performance in identifying roads, with the main difference being their depiction of road details. Specifically, the similarity between dry river channels and roads is high. Both the LinkNet and BiSeNet methods extracted obvious dry river channels, and using the ASUNet algorithm can, to some extent, avoid the influence of river channels (I). For situations where the background and road information are highly similar (II) road information is similar to the height of village houses; III road information is similar to the height of surrounding farmland), this algorithm can still extract complete and clear roads well. In addition, in the suburb scenario (IV and V), the algorithm can even recognize unmarked road segments with high continuity and completeness. In more complex urban scenes (VI), especially when buildings and roads are extremely similar, the proposed roads have clearer boundaries, which are more in line with people’s visual recognition of roads.

It should be noted that in this set of prediction results, there are many details on the road that are not included in the label (taking the fourth line as an example). After inspection and verification, the information provided is correct. The reason is that the Zhouqu Road Dataset is a self-built dataset, and there were slight omissions in road markings. Therefore, the provided information effectively supplements the shortcomings in the label, and the extraction is accurate.

The experimental results demonstrate that ASUnet outperforms several mainstream semantic segmentation networks, such as BiSeNet, LinkNet, and Unet, on the DeepGlobe Road Dataset and the self-constructed Zhouqu Road Dataset. Superior performance is achieved in terms of accuracy, recall, F1 score, IoU, and mean IoU. Additionally, ablation studies further validate the effectiveness of the strip and atrous convolution modules.

## 4. Discussion

In this section, an ablation study is conducted on the ASUNet model. Experiments are performed on the baseline model UNet, with the following configurations: UNet + ResNet-50, UNet + ResNet-50 + Atrous Convolution, and UNet + ResNet-50 + Atrous + Strip. This series of experiments aims to investigate which specific components of the model play a crucial role in improving the performance of road extraction.

### 4.1. Ablation Experiment

To verify the effectiveness of the proposed atrous convolution module and strip convolution module, this paper conducted ablation experiments using the UNet model as the base model. The results of the ablation experiment are shown in Table 3.

In the road detection and segmentation tasks of the Zhouqu Road Dataset and DeepGlobe Datasets, the performance of each model not only reflects the value of module iteration but also highlights the unique advantages of the ASUNET (U: UNet basic segmentation network + R: ResNet-50 feature extraction network + A: Atrous Convolution + S: Strip strip feature module) model proposed in this paper. On two datasets, similar ablation patterns were demonstrated, from the basic model “U (UNet)” to “U + R (Unet + ResNet-50)”, “U + R + A (Unet + ResNet-50 + atrous convolution)”, and “U + R + S (Unet + ResNet-50 + Strep)”. It can be seen that the ResNet-50 module, as the core foundation, effectively extracts key features with its deep residual structure, solving the problems of missed detection of roads and low segmentation accuracy in basic UNet (recall increased from 0.6545 to 0.7908 on the Zhouqu Road Dataset, and road IoU increased from 0.49 to 0.5842). On the DeepGlobe Dataset, recall increased from 0.6887 to 0.8430, and road IoU increased from 0.5375 to 0.6113. The Astrous module further optimized the accuracy of road recognition by expanding the receptive field to preserve road details (precision reached 0.7017 and road IoU reached 0.5947 on the Zhouqu Road Dataset, and precision reached 0.6991 and road IoU reached 0.6197 on the DeepGlobe Dataset). The Strip module relies on its adaptability to the linear structure of roads, improving the overall classification accuracy (F1 value of 0.7380 on the Zhouqu Road Dataset and 0.7357 on the DeepGlobe Dataset). ASUNET achieved collaborative integration of segmentation, feature extraction, detail preservation, and structural adaptation capabilities by integrating the four modules of Unet + ResNet-50 + atrous convolution + Strip, demonstrating significant technical highlights and practical value.

From the perspective of core indicators, the ASUNET indicators in both datasets are slightly lower than “U + R + A (Unet + ResNet-50 + dilated convolution)”, but they have three key advantages: Firstly, they have outstanding accuracy, with the highest precision among all models, which is due to ResNet-50’s deep feature filtering, dilated convolution’s capture of road and non-road boundary details, and the Strip module’s accurate recognition of road strip shapes—in complex scenarios such as blurred road and non-road boundaries and vegetation occlusion, they can minimize the error of “non-road misjudgment as road” to the greatest extent possible, which is crucial for “avoiding engineering decision deviations caused by misjudgment” in practical applications (such as road repair and path planning). Secondly, ASUNET has stronger module compatibility and scalability. Compared with models with single functional modules (such as “U + R + A”, focusing on optimizing road segmentation details through dilated convolutions, and “U + R + S”, focusing on improving overall classification through strips), ASUNET integrates the dual mechanism of preserving details through dilated convolutions and adapting to the linear structure of strips. It can not only retain precise focus on road categories but also take into account global awareness of complex backgrounds, making it more suitable for the needs of “road and multi-category environment interaction” in real scenes (such as distinguishing roads from surrounding buildings and farmland). Thirdly, it has better performance stability, with accuracy on par with “U + R + A”, and all indicators are at a high level (without obvious shortcomings), avoiding the problem of decreased road segmentation accuracy caused by “U + R + S” focusing on linear structure classification of strips, and demonstrating a more balanced comprehensive ability.

In addition, the design idea of ASUNET is innovative in technology—it breaks the “functional limitation of a single module” by integrating four complementary modules of Unet, ResNet-50, void convolution and Strip, and provides more space for subsequent optimization. The small fluctuations in current indicators are more likely to be the adjustable direction of module integration strategies (such as the feature weight distribution of Strip and void convolution, the hierarchical interaction mode of ResNet-50 and Unet), rather than defects of the model architecture. If the collaborative mechanism between modules is further optimized (such as enhancing the detailed guidance effect of dilated convolution on road features and improving the adaptation accuracy between Strip modules and road linear structures), ASUNET is expected to further improve recall and segmentation accuracy while maintaining high accuracy, becoming a high-performance model more suitable for complex mountainous road scenes.

**Figure 8:**
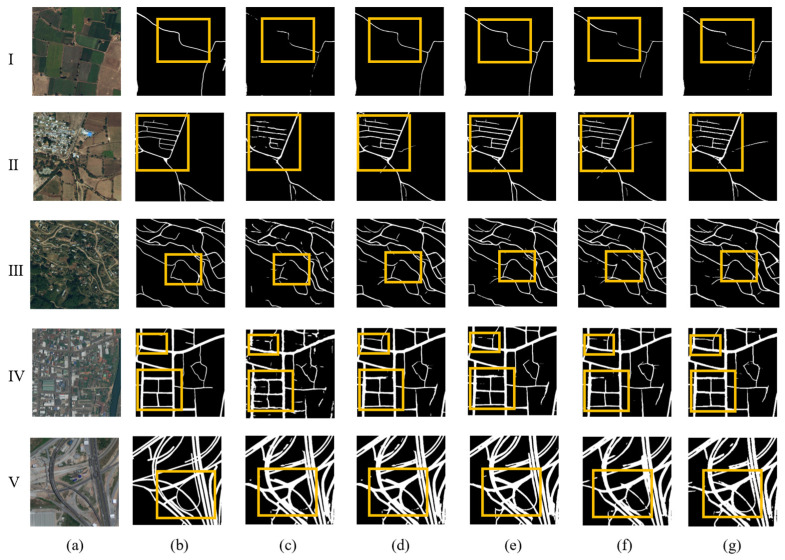
Visualized experimental results on the DeepGlobe test set. (**a**) Remote sensing imagery; (**b**) road labels; (**c**)Unet; (**d**) Unet + ResNet-50; (**e**) Unet + ResNet-50 + Atrous; (**f**) Unet + ResNet-50 + Strip; (**g**) Unet + ResNet-50 + Atrous + Strip (ASUNet).

From the visualization ablation experiment results of the DeepGlobe Dataset in Figure 8, it can be seen that with the addition of different modules, road information extraction is continuously optimized. The Astrous convolution module can combine the features of multi-scale receptive fields to better learn contextual information, enhance semantic representation, and more effectively identify roads obscured by vegetation (I). Due to its emphasis on the shape of roads, the strip convolution module can refine the extraction results, improve connectivity, and better distinguish roads with similar colors and backgrounds (III). In addition, with the addition of the strip module, the misclassification that occurs near buildings has gradually improved (II, IV), and we can identify roads that we believe are not labeled in the label but are correct and actually exist, indicating that these two modules have played a role in correctly classifying roads near buildings. In complex urban roads (V), the identification of overpasses, as shown in the figure, has also been well applied for the continuous identification of narrow roads. It can be seen that the atrous convolution module and strip convolution module designed in this paper effectively enhance the road continuity of small roads and improve road segmentation in complex environments.

**Figure 9:**
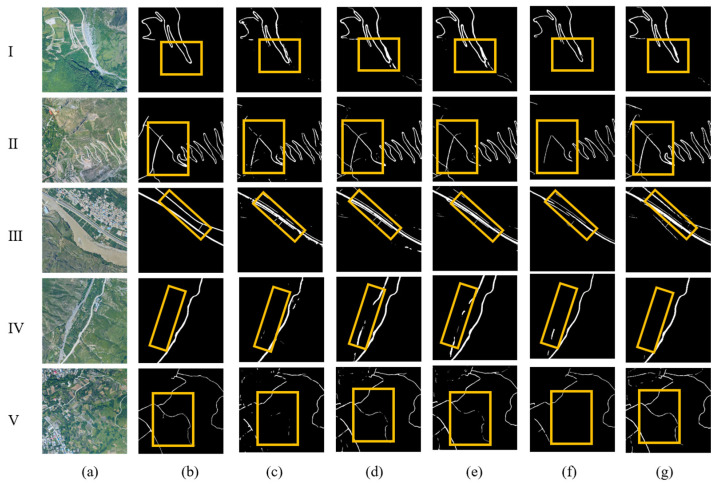
Visualized experimental results on the Zhouqu test set. (**a**) Remote sensing imagery; (**b**) road labels; (**c**) Unet; (**d**) Unet + ResNet-50; (**e**) Unet + ResNet-50 + Atrous; (**f**) Unet + ResNet-50 + Strip; (**g**) Unet + ResNet-50 + Atrous + Strip (ASUNet).

From the Zhouqu Road Dataset in Figure 9, it can be seen that in rural areas (I, II), the roads formed through processes, such as adding grids, atrous convolution, and strip convolution, become clearer and more complete, effectively eliminating the impact of bare soil and bare river channels on road information extraction. At the edge of the city (III), the addition of modules enables more effective identification of roads similar to buildings. It can effectively avoid the impact of dry river channels on road extraction (IV). In the case of narrow roads with complex backgrounds, our proposed model can effectively extract weak road information (V).

### 4.2. Model Analysis

We conducted model parameter analysis on four models, BiSeNet, LinkNet, UNet, and ASUnet, in terms of inference speed, Parameter Quantity, and FLOPs [43]. From the perspective of performance indicators and design logic (Table 4), the architecture design of ASUnet clearly focuses on the core accuracy requirements of road extraction tasks. Although its 180.81 M parameter count far exceeds the other three models (BiSeNet 13.39 M, LinkNet 11.54 M, UNet 31.04 M), reaching 13.5 times that of the lightweight road extraction model BiSeNet, this design choice is actually a targeted breakthrough for the difficult problems in road extraction scenarios—the introduction of complex modules, such as multi-scale attention and dense connections, which are precisely aimed at accurately optimizing key aspects of traditional models, such as “narrow roads” and “road edge restoration under tree/building occlusion”. From the original design intention, the model aims to improve the detail accuracy and topological integrity of road segmentation, especially to meet the road extraction requirements of complex scenes in high-resolution remote sensing images.

From the perspective of computational efficiency, the computational complexity of ASUnet, 138.59 GFLOPs, is only slightly higher than that of UNet in the same scenario (124.66 GFLOPs), indicating that the introduction of complex modules has not led to an exponential increase in computational complexity. In the “high-precision oriented” road extraction model, this computational control has already demonstrated certain design considerations. Although the inference speed of 8.78 FPS is only 31.9% of UNet and 4.4% of BiSeNet, it needs to be objectively considered in conjunction with its core application scenarios—such models are more targeted at offline road extraction tasks that require strict accuracy, such as high-precision road map construction and road asset refinement survey. The compromise in speed indicators can be compensated for by high-performance computing resources in the backend. The complex modules designed for road features in its architecture still provide valuable technical exploration directions for solving the accuracy bottleneck in the field of road extraction.

### 4.3. Discussion on Module Effectiveness

This study proposes a novel network model, ASUnet, for extracting roads from satellite imagery. The model employs an encoder–decoder architecture, augmented with strip convolution modules and atrous convolution modules, to enhance the learning of road features. The strip convolution module captures long-range contextual information through four directional strip convolutions, thereby reducing the interference of unrelated regions on feature learning. The atrous convolution module expands the receptive field via atrous convolutions and integrates multi-scale features through skip connections, preserving low-level feature details.

The accuracy of UNet + ResNet50 is better than that of UNet. The reason is that ResNet-50 provides powerful feature extraction capabilities that can capture richer semantic information. Feature fusion combines the detail preservation capability of UNet with the semantic extraction capability of ResNet-50. The residual learning mechanism alleviates the gradient vanishing problem in deep networks, enabling the model to learn more complex feature representations. On this basis, Atrous convolution (UNet + ResNet-50 + Atrous convolution) is added, which further improves the accuracy. The reason is that the dilated convolution expands the receptive field of the convolution kernel, allowing each convolution kernel to cover a larger image region, capture a wider range of contextual information, and extract features of different scales—an approach highly effective for capturing roads of varying widths and shapes, maintaining road connectivity, and addressing issues like noise and shadow occlusion in complex scenes, thus enhancing the model’s robustness.

Continuing to introduce the strip module (UNet + ResNet-50 + Atrous convolution, also known as ASUNET), the complexity of the Zhouqu Dataset enables strip convolution to better handle noise and occlusion, thereby improving accuracy and precision. However, in some cases, excessive smoothing or insufficient detection of small roads may be introduced, resulting in a lack of further improvement in recall and F1 score. The regularity and high resolution of the DeepGlobe Dataset enable strip convolution to better capture the directionality and connectivity of roads, thereby improving F1 score, road IoU, and mIoU. However, in some cases, strip convolution may be more sensitive to noise, leading to a lack of further improvement in precision and recall. Overall, for unknown road conditions, adding a strip module to the model would have better performance. The visualization results show that adding the module will improve road connectivity.

While strip convolution excels at capturing multi-directional linear road features, it struggles with sharp/tortuous road segments (e.g., Zhouqu’s mountain winding roads). Due to fixed kernel orientation and coverage, it tends to fragment curved road features, causing segmentation breaks or misclassification. In ASUNet, the global semantics of atrous convolution partially offset this limitation, but further optimization (e.g., adjusting kernel orientation) is still needed for extremely curved segments to enhance robustness. Taken together, these two modules complement each other: atrous convolution enlarges the receptive field and captures global semantics, while strip convolution enhances sensitivity to linear structures across multiple directions. Their integration enables ASUNet to achieve both semantic completeness and structural continuity.

Although this study integrates strip convolution and atrous convolution for road extraction, achieving favorable performance in scenarios with complex backgrounds, several limitations of the technique should be noted. First, when dealing with extremely narrow roads or roads with continuous sharp bends, the model tends to produce discontinuous results in road recognition. Second, for more structurally complex road types (e.g., highways and overpasses), the model fails to fully learn the features of such roads due to insufficient training samples, leaving room for improvement in prediction accuracy. In addition, the proposed model incorporates an extensive set of training parameters, which inherently results in relatively low training efficiency. Consequently, the model exhibits significant room for optimization with respect to training efficiency.

## 5. Conclusions

The main contributions of this paper are as follows:

(1) Development of the Zhouqu Road Dataset. Based on high-resolution aerial imagery, this study constructs the Zhouqu Road Dataset, which encompasses rural roads, urban roads, and roads in urban–rural fringe areas. This dataset serves as a high-quality benchmark, representing the typical road features in mountainous regions of western China.
(2) Innovative Improvements of the ASUnet Model. ASUnet introduces the atrous and strip convolution modules to significantly enhance the precision and robustness of road extraction. The atrous convolution module expands the receptive field to capture global contextual information, while the strip convolution module captures long-range features in multiple directions, minimizing the interference from unrelated regions. Experimental results indicate that the incorporation of these modules effectively balances precision and recall, thereby improving the F1 score and mean IoU performance. Notably, the model demonstrates superior adaptability in extracting roads obscured by vegetation or with colors similar to the background. Comparative experiments among ResUnet, ResUnet with atrous convolution modules, and ResUnet with both atrous and strip convolution modules further validate the effectiveness of these modules in road image segmentation.

Despite its strong performance in road extraction, ASUnet still faces challenges in distinguishing between roads and rivers, as well as between dirt roads and dried-up riverbeds, due to their similar shapes and textures. This highlights the model’s limitations in differentiating geographical features with high similarity. However, ASUnet’s robust feature learning and detail-capturing capabilities make it not only suitable for road extraction but also applicable to other similar fields, such as vascular extraction in medical imaging and river extraction in natural environments.

In summary, ASUnet offers an effective solution for road extraction from remote sensing imagery, demonstrating high adaptability in complex backgrounds and low-contrast scenarios.

## Figures and Tables

**Table 1:** McNemar tests results for the classification of the model.

Dataset	Model	$f_{12}$	$f_{21}$	$X^{2}$	*p*
Zhouqu Road	ASUnet vs. BiSeNet	1,591,930	1,202,623	54,233.776	<0.001
	ASUnet vs. LinkNet	3,995,596	1,361,330	1,295,398.161	<0.001
	LinkNet vs. BiSeNet	3,292,608	1,047,649	1,161,183.870	<0.001
DeepGlobe Road	ASUnet vs. BiSeNet	18,490,490	10,083,152	2,473,724.401	<0.001
	ASUnet vs. LinkNet	13,996,343	9,425,661	891,944.379	<0.001
	LinkNet vs. BiSeNet	13,495,452	9,658,796	635,733.088	<0.001

$f_{12}$: the first one correctly but the second one incorrectly, $f_{21}$: the first one incorrectly but the second one correctly.

**Table 2:** Comparison of experimental results of different models for the Zhouqu Road Dataset and the DeepGlobe Road Dataset.

Dataset	Model	Accuracy	Precision	Recall	F1 Score	Road IoU	mIoU
Zhouqu Road Dataset	BiSeNet	0.9708	0.4545	0.615	0.5227	0.3538	0.6621
	LinkNet	0.9831	0.6491	0.7608	0.7005	0.539	0.7609
	**ASUnet**	**0.9859**	**0.7026**	**0.7905**	**0.7292**	**0.5738**	**0.7832**
DeepGlobe Road Dataset	BiSeNet	0.9748	0.7252	0.6339	0.6765	0.5111	0.7426
	LinkNet	0.9762	0.7210	0.7000	0.7103	0.5508	0.7632
	**ASUnet**	**0.9793**	**0.7722**	**0.7134**	**0.7417**	**0.5894**	**0.7841**

**Table 3:** Ablation results for the Zhouqu Road Dataset and the DeepGlobe Road Dataset.

Dataset	Model	Accuracy	Precision	Recall	F1 Score	Road IoU	mIoU
Zhouqu Road Dataset	U	0.9825	0.6681	0.6545	0.6586	0.49	0.7361
	U + R	0.9852	0.6862	0.7908	0.7376	0.5842	0.7828
	U + R + A	0.9859	0.7017	**0.7958**	**0.7458**	**0.5947**	**0.7901**
	U + R + S	0.9921	0.6997	0.7808	0.7380	0.5848	0.7851
	U + R + A + S **(ASUNET)** **our model**	**0.9859**	**0.7026**	0.7905	0.7292	0.5738	0.7832
DeepGlobe Road Dataset	U	0.9740	0.7101	0.6887	0.6992	0.5375	0.7554
	U + R	0.9765	0.6898	0.8430	0.7588	0.6113	0.7934
	U + R + A	0.9772	0.6991	**0.8452**	**0.7652**	**0.6197**	**0.7980**
	U + R + S	0.9750	0.6869	0.7921	0.7357	0.5820	0.7780
	U + R + A + S **(ASUNET)** **our model**	**0.9793**	**0.7722**	0.7134	0.7417	0.5894	0.7711

U: Unet; R: ResNet-50; A: Atrous convolution; S: Strip.

**Table 4:** Model efficiency analysis table.

Model	Inference Speed (FPS)	Parameter Quantity (M)	FLOPs (GFLOPs)
BiSeNet	199.29	13.39	14.862
LinkNet	166.15	11.54	4.955
UNet	27.57	31.04	124.66
ASUnet	8.78	180.81	138.59

## Data Availability

The DeepGlobe Road Dataset is available at http://deepglobe.org/challenge.html (accessed on 1 February 2026).
